# Single-cell profiling reveals a reduced epithelial defense system, decreased immune responses and the immune regulatory roles of different fibroblast subpopulations in chronic atrophic gastritis

**DOI:** 10.1186/s12967-025-06150-w

**Published:** 2025-02-04

**Authors:** Lin Lin, Tingxuan Huang, Lizhi Li, Yang Lin, Feng Chen, Ziyi Zheng, Jie Zhou, Yizhe Wang, Weihao You, Yujie Duan, Yawen An, Shiwei He, Weimin Ye

**Affiliations:** 1 Institute of Population Medicine, School of Public Health, Fujian Medical University, 1 Xuefu North Road, Fuzhou, 350122 China; 2 Department of Gastroenterology, Fujian Medical University Union Hospital, Fuzhou, 350001 China; 3 Fujian Clinical Research Center for Digestive System Tumors and Upper Gastrointestinal Diseases, Fuzhou, 350001 China; 4 Department of Pediatric Surgery, Shengli Clinical Medical College of Fujian Medical University, Fuzhou, 350001 China; 5 Key Laboratory of Ministry of Education for Gastrointestinal Cancer, Fujian Medical University, Fuzhou, 350122 China; 6 Department of Medical Epidemiology and Biostatistics, Karolinska Institutet, Stockholm, 17177 Sweden; 7 Department of Pediatric Surgery, Fujian Medical University Union Hospital, Fuzhou, 350001 China

**Keywords:** Single-cell RNA sequencing (scRNA-seq), CAG, Detoxification, Immune dysfunction, Fibroblast, T-cell exhaustion

## Abstract

**Purpose:**

To identify key cellular changes and molecular events in atrophic mucosa, we aimed to elucidate the molecular mechanisms driving the occurrence of chronic atrophic gastritis (CAG).

**Methods:**

We used single-cell RNA sequencing (scRNA-seq) to characterize changes in the epithelial state and tissue microenvironment associated with CAG. The molecular changes were identified by comparing differentially expressed genes (DEGs) between the two mucosa states. Gene Ontology (GO) pathway enrichment analysis was used to explore the potential functional changes in each cell subtype in atrophic mucosa. Gene set score analysis was conducted to compare the functional roles of different fibroblast subtypes and functional changes in cell subtypes between the CAG and control groups. Metabolic analysis was performed to compare the metabolic activity of *C1Q*^+^ macrophages under different conditions. NichNet analysis was used to analyze the regulatory relationships between *CCL11*^+^*APOE*^+^ fibroblasts and *C1Q*^+^ macrophages and between *CCL11*^+^*APOE*^+^ fibroblasts and *CD8*^+^ effector T cells. Transcription factor (TF) analysis was performed to determine the transcription status of different T-cell subtypes in atrophic and normal mucosa.

**Results:**

We generated a single-cell transcriptomic atlas from 3 CAG biopsy samples and paired adjacent normal tissues. Our analysis revealed that chief cells and parietal cells exhibited a loss of detoxification ability and that surface mucous cells displayed a reduced antimicrobial defense ability in CAG lesions. The mucous neck cells in CAG lesions showed upregulation of genes related to cell cycle transition, which may lead to aberrant DNA replication. Additionally, cells with the T exhaustion phenotype infiltrated under CAG condition. *C1Q*^*+*^ macrophages exhibited reduced phagocytosis, downregulated expression of pattern recognition receptors and decreased metabolic activity. NichNet analysis revealed that a subpopulation of *CXCL11*^+^*APOE*^+^ fibroblasts regulated the inflammatory response in the pathogenesis of atrophic gastritis. *APSN*^+^*CXCL11*^+^*APOE*^+^ fibroblasts were found to be associated with gastric cancer (GC) development.

**Conclusions:**

The main goal of this study was to comprehensively elucidate the cellular changes in CAG lesions. We observed an immune decline in the mucosal microenvironment during the development of CAG, including a reduced immune response of *C1Q*^+^ macrophages, reduced cytotoxicity of T cells, and increased infiltration of exhausted T cells. Specifically, we demonstrated that different epithelial subtypes aberrantly express genes related to susceptibility to external bacterial infection and aberrant cell cycle progression. Our study provides new insights into the functions of epithelial changes and immune alterations during the development of CAG.

**Supplementary Information:**

The online version contains supplementary material available at 10.1186/s12967-025-06150-w.

## Introduction

Chronic atrophic gastritis (CAG) is a prevailing chronic digestive system condition that is important because it is considered the main precancerous condition of gastric cancer (GC), according to Correa’s cascade model [1]. Patients with CAG have an increased risk of GC [2]. Normal mucosa can develop into atrophic mucosa, which is characterized destructive cellular changes, including a decrease in or disappearance of parietal cells, the production of pepsinogen-producing chief cells and the infiltration of immune cells to act on damaged epithelial cells [3, 4]. Thus, an in-depth understanding of the main cellular changes in atrophic gastric mucosa could provide new insights for further timely intervention and the discovery of new diagnostic targets.

The application of single-cell RNA sequencing (scRNA-seq) technology has allowed researchers to determine the phenotypic changes in different cell types in GC tissues, resulting in a comprehensive and accurate analysis of individual cells in the tumor microenvironment. Heterogeneous cell states are observed at the end of tumorigenesis [5–7]. Multiple-stage carcinogenesis studies revealed that several pathways were enriched in epithelial cells across different lesions: the tumor necrosis factor signaling pathway and mineral absorption pathway in CAG lesions, metabolic pathways in intestinal metaplasia (IM) lesions, and cell proliferation-related pathways in early gastric cancer lesions [8]. Recently, several key events, including high levels of immune exhaustion in T cells and enrichment of oxidative phosphorylation in intestinal stem cells, have been identified by single-cell studies of IM lesions [9]. Compared with those in normal mucosa, the expression of several genes has been shown to be upregulated in CAG [10]. However, little is known about the changes in expression of different cell types in CAG, and comprehensive assessments of the transcriptional transition from normal mucosa to atrophic mucosa are lacking. A previous study revealed that interleukin 13 is secreted by multiple subsets of immune cells and acts directly on the gastric epithelium to promote neck cell expansion and metaplasia [11]. This study emphasizes the importance of identifying cytokine-producing immune cell populations that are involved in the progression of atrophic mucosa. Moreover, persistent *Helicobacter pylori (H. pylori)* infection is an important pathogenic mechanism, and *H. pylori* infection is believed to alter cell infiltration in the gastric mucosa [12]. Gastric stromal cells secrete angiogenesis-regulating factors to promote bacterial infection-associated pathology. VacA, an immunomodulatory toxin, can inhibit the activation and proliferation of immune cells and suppress the function of macrophages, leading to impaired immune clearance and persistent *H. pylori* infection [13]. Infection with *H. pylori* increases host ANGPTL4 expression, which reshapes the gastric environment by promoting the infiltration of T regulatory cells (Tregs) and decreasing neutrophil accumulation in the infected gastric mucosa, directly leading to progression of gastritis and bacterial persistence [14]. In addition to immune alterations, gastric epithelial cells are believed to be major effector cells that are infected by *H. pylori* and induce a biased inflammatory response via the secretion of proinflammatory molecules [15]. Single-cell profiling of non-atrophic gastritis, IM, and GC samples has revealed different transcriptional features related to *H. pylori* infection [16], however, the cellular functional changes in CAG patients still need to be investigated.

Here, to clarify the altered phenotypes of epithelial cells, immune cells and stromal cells involved in CAG lesions, we utilized scRNA-seq to characterize the transcriptional heterogeneity of CAG. Through this study, we further elucidated how different immune cells, fibroblasts, and epithelial cells acquire new gene expression programs during the development of CAG. Our findings provide new insights into the development of new therapeutic strategies by reinvigorating the exhausted phenotype of adaptive immune cells, promoting the phagocytosis of innate immune cells and rescuing the detoxification and antimicrobial abilities of epithelial cells.

## Materials and methods

### Patient recruitment and sample collection

This study was approved by the Biomedical Research Ethics Review Committee of Fujian Medical University. All participants provided their informed consent before participating in the study. Patients were diagnosed by endoscopists with CAG C2 based on the Kimura–Takemoto classification system. Three pairs of CAG lesions located in the gastric body and neighboring normal tissues were collected via biopsy from the CAG patients prior to any treatment. All the clinical characteristics of the participants are shown in Additional file 6: Table S1.

### Single-cell suspension preparation

The collected gastric mucosa samples were stored in tissue storage solution (MACS, No.130-100-008) and processed into single-cell suspensions within 24 h. The tissue samples were minced with Iris scissors into small pieces and dissociated using collagenase II (Sigma, V900892-100MG), collagenase I (Sigma, V900891-100MG) and DNase I (Sigma, 9003-98-9) at 37 °C for 1 h. The digested sample was further filtered through a 40 mm cell strainer (Falcon^®^, No. 352340). After centrifugation, the supernatant was removed, and the remaining cell pellets were resuspended in red blood cell lysis buffer (Solarbio, R1010) for 5 min to lyse the erythrocytes. The lysis reaction was terminated with RPMI 1640 supplemented with 10% FBS. The cell count and viability were determined via a fluorescence cell analyzer (Countstar^®^ Rigel S2) with AO/PI reagent (Countstar^®^, RE010212). Fresh cells were washed twice in RPMI 1640 medium supplemented with 10% FBS and then resuspended at 1 × 10^6^ cells/mL in 1× PBS supplemented with 0.04% BSA. Finally, 10,000 to 12,000 cells were added for library construction and sequencing.

### Library construction and sequencing

scRNA-seq libraries were prepared via a SeekOne^®^ Digital Droplet Single Cell 3’ library preparation kit (SeekGene, No. K00202). Briefly, an appropriate number of cells were mixed with reverse transcription reagent and then added to the sample well on a SeekOne^®^ chip. Subsequently, barcoded hydrogel beads (BHBs) and partitioning oil were dispensed into the corresponding wells separately in the chip. After the emulsion droplets were generated, reverse transcription was performed at 42°C for 90 minutes, and the mixture was then inactivated at 80°C for 15 minutes. Next, cDNA was purified from the broken droplets and amplified via PCR. The amplified cDNA product was then cleaned, fragmented, end repaired, A-tailed and ligated to a sequencing adaptor. Finally, indexed PCR was performed to amplify the DNA representing the 3’ polyA region of the expressed genes, which also contained the cell barcode and unique molecular index. The indexed sequencing libraries were cleaned with SPRI beads, quantified by quantitative PCR (KAPA Biosystems KK4824) and then sequenced on a NovaSeq™ X Plus instrument with a PE150 read length.

### Single-cell RNA-seq data analysis

We used SeekOne^®^Tools to analyze the original sequence data, which were subsequently aligned to the human GRCh38 genome reference genome to construct a gene expression matrix. These data were subsequently analyzed via the R package Seurat v5.0.3 for cell quality control [17]. The cells were removed on the basis of the expression levels of mitochondrial genes, ribosome genes and hemoglobin genes. The remaining cells were further subjected to dimensionality reduction and integration analysis. We used the IntegrateData function to integrate the 6 data files. We standardized each data matrix file by using the SCTransform function. RunHarmony was used to correct batch effects produced by possible experimental variables. Finally, we conducted dimensionality reduction on the integrated data and identified cell subpopulations via the FindClusters function by setting different resolutions. The cell types were annotated manually on the basis of canonical cell markers, including markers for epithelial cells (*CDH1*, *EPCAM*), T cells (*CD3D*, *CD3E*), myeloid cells (*CD74*, *CD14*, *CD68*), endothelial cells (*PLVAP*, *PECAM1*), fibroblasts (*DCN*, *POSTN*), mast cells (*KIT*, *TPSAB1*), pericytes (*RGS5*), B cells (*CD79A*, *BANK1*, *MS4A1*) and plasma cells (*MZB1*, *JCHAIN*).

### Functional enrichment analysis

Marker genes were identified via the “COSG” function with default parameters [18]. This analysis aimed to identify changes in gene expression between two conditions or cell types. The results of the differential gene expression analysis were further subjected to functional enrichment analysis via the clusterProfiler package [19]. The gene lists generated from the analysis were evaluated for enrichment in biological process (BP) terms from the Gene Ontology (GO) Resource [20].

### Definition of cell scores and signatures

To evaluate the potential functionality of the cell cluster of interest, we computed signature scores via the “AddModuleScore” function in Seurat to assess cluster identity. Scores were calculated by binning signature genes in 12 bins according to the average expression. The signature’s average expression was corrected by subtracting the aggregated expression of 100 randomly selected genes from the same bin. The gene lists used to define signature scores are listed in Additional file 8: Table S3 and Additional file 9: Table S4. The list of genes used for generating the signature score of atrophic gastritis susceptibility genes was downloaded from the Harmonizome 3.0 database [21].

### NicheNet analysis

We used the NicheNet method to conduct a detailed analysis of the predicted ligands that drive the expression changes in the target cell subpopulations [22]. The target cell subpopulations of interest included *C1Q*^+^ macrophages and *CD8*^+^ effector T-cell subpopulations. The sender cell subpopulation of interest was *CCL11*^+^*APOE*^*+*^ fibroblasts. We first classified target cell populations by the observed conditions (CAG and control). Then, gene sets of interest within each target cell subpopulation were first identified via the FindMarkers function. Genes with log2FC > 0.25 and adjusted p value < 0.05 were further filtered. All expressed genes in the target cells were used as the background for the genes. Genes were considered expressed when they had nonzero values in at least 5% of the cells in a given cell type.

### Metabolic pathway analysis

We used the “scMetabolism” package to assess metabolic activity at the single-cell level [23]. This method was performed with preloading of the Kyoto Encyclopedia of Genes and Genomes (KEGG) database to evaluate the metabolic gene sets. First, a cell expression matrix was prepared. The metabolic activity of each cell was quantified via the Vision algorithm implemented in the “scMetabolism” package. Visualization of the results was achieved via the DotPlot.metabolism and BoxPlot.metabolism functions. The difference in activity between groups was calculated via a two-sided Wilcoxon rank-sum test.

### Transcription factor (TF) analysis

We used the “Dorothea” package to infer TF activity between different T-cell subtypes and the TF activity of each T-cell subtype in different mucosal states. This algorithm constructed regulons on the basis of the mRNA expression levels of each TF from a manually curated database, along with the expression level of its direct targets. TF regulons were generated via the ‘dorothea_regulon_human’ function, and ‘A’, ‘B’ and ‘C’ high-confidence TFs were selected for further analysis. TF activity represents the transcriptional state of its direct targets. The Viper score, mean and standard deviation were calculated for each group. Highly variable TFs were ranked on the basis of the variance of their corresponding viper scores.

### Recombinant CCL2 treatment

For recombinant CCL2 protein treatment, the THP-1 and U937 cell lines were grown as usual and plated into 24-well plates. The THP-1 and U937 cell lines were treated with lipopolysaccharide (LPS) (0.5 µg/mL, MCE) for 2 h, after which recombinant CCL2 protein (160 ng/mL, SinoBiological, Cat: 10134-H08Y) was added to the medium. At the indicated time points (72 h), the cells were harvested for mRNA analysis.

### Quantitative real-time RT‒PCR

Total RNA was extracted via Total RNA Extraction Reagent (Yeasen, Cat: 10606ES60). RNA was quantified via a NanoDrop One instrument (Thermo Scientific, USA). Reverse transcription was performed via the addition of 1 µg of total RNA with 1st Strand cDNA Synthesis SuperMix for qPCR (Yeasen, Cat:11141ES60). The mRNA expression level was determined via quantitative real-time PCR with SYBR Green Master Mix (Yeasen, Cat:11185ES08) and a Celemetor Real-Time PCR System (Yeasen, China). Human glyceraldehyde-3-phosphate dehydrogenase (GAPDH) was used as an internal control for RNA integrity.

*ASPN*-F CTCTGCCAAACCCTTCTTTAGC.

*ASPN*-R CGTGAATAGCACTGACATCCAA.

*CCL4*-F CTGTGCTGATCCCAGTGAATC.

*CCL4*-R TCAGTTCAGTTCCAGGTCATACA.

*SOD2*-F GCTCCGGTTTTGGGGTATCTG.

*SOD2*-R GCGTTGATGTGAGGTTCCAG.

*GAPDH*-F GGAGCGAGATCCCTCCAAAAT-3’.

*GAPDH*-R GGCTGTTGTCATACTTCTCATGG-3’.

### Multiplex immunohistochemistry (IHC)

The slides were baked for approximately 1 h at 60 °C, followed by deparaffinization and rehydration. Then, the slides were subjected to antigen retrieval via Tris-EDTA (Solarbio, China), blocked with 10% goat serum (Solarbio, China), and immunostained with primary antibodies, which included antibodies against ASPN (Sigma, 1:100), DCN (Proteintech, 1:1000), CSF1 (Diagbio, 1:100), and CSF1R (ABclonal, 1:100), followed by incubation with a fluorescent secondary antibody or an HRP-conjugated secondary antibody (Abcam, goat anti-rabbit Alexa Fluor 488, goat anti-mouse Alexa Fluor 647, and Panovue, goat anti-rabbit Alexa Fluor 555, diluted 1:500). Once all the markers were labeled, the slides were counterstained with DAPI (Panovue), mounted in antifade mounting medium, and examined via a Leica TCS SP5 confocal microscope.

### Public dataset

The key phrase “gastritis” was used as a search term in the GEO database (https://www.ncbi.nlm.nih.gov/geo/). As a result, three datasets (GSE130823, GSE191275 and GSE153224) were included for ASPN expression analysis. All the data are accessible online free of charge. The GSE130823 dataset was based on the GPL17077 platform [Agilent-039494 SurePrint G3 Human GE v.2 8 × 60 K Microarray 039381 (Probe Name version)], which comprises 47 gastritis, 17 low-grade intraepithelial neoplasia (LGIN), 14 high-grade intraepithelial neoplasia (HGIN), and 16 GC samples. The GSE153224 dataset was based on the GPL20301 platform [Illumina HiSeq 4000 (*Homo sapiens*)], which comprises 5 CAG and 5 nonchronic gastritis (NAG) samples. The GSE191275 dataset was based on the GPL20301 platform [Illumina HiSeq 4000 (*Homo sapiens*)], which comprises 10 NAG samples and 10 GC samples.

### Statistical analysis

All the statistical analyses were conducted via R software (https://www.R-project.org/) v4.2.0. Detailed descriptions of the statistical methods used are provided in the figure caption section.

### Code availability

Code for scRNA-seq data analysis in this study was performed according to the tutorial on the Seurat website: https://satijalab.org/seurat/articles/pbmc3k_tutorial. Additional code described in detail throughout the Methods and is available on GitHub (https://github.com/zeroLB/CAG_code). All the analyses and plots have been made on R (v4.0.4) environments, using the third-party libraries mentioned in the Methods.

## Results

### Single-cell transcriptional profiling dissects the cellular ecosystem of CAG

**Fig. 1:**
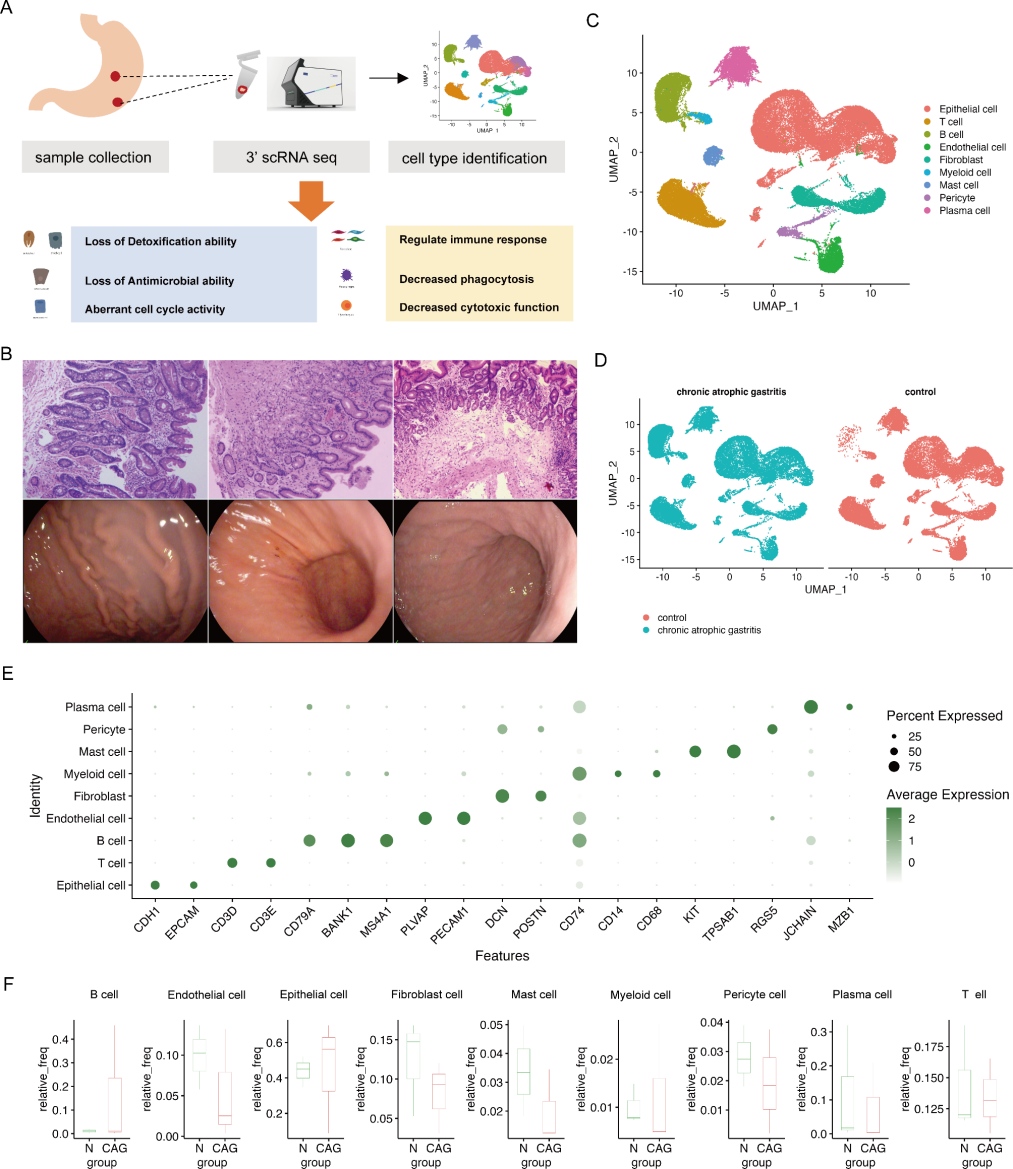
The landscape of the microenvironment in CAG at single-cell resolution. (**A**) Graphical summary of the experimental design. Paired atrophic mucosa and normal mucosa tissues were processed via single-cell 3’ RNA sequencing for transcriptomic analysis. (**B**) The clinical manifestations and HE staining of atrophic mucosa. (**C**) UMAP visualization of major cell types, showing cell types by color. (**D**) UMAP visualization of the cell type distribution according to disease condition. (**E**) Dot plot showing the expression of marker genes for each cell type. (**F**) The relative abundance of the major cell lineages in the CAG and control samples.

To determine the heterogeneous cellular profile of chronic atrophic lesions, we collected three pairs of CAG biopsies located in the gastric body and neighboring normal mucosa (Additional file 5: Table S1). For each biopsy, we prepared single-cell solutions without the selection of certain cell types and applied the SeekOne platform to generate the scRNA-seq data. After low-quality cells were removed, a total of 62,542 cells were retained for subsequent analysis, which yielded a median of 2,431 genes detected per cell (Fig. 1A). The number of cells from each biopsy is provided in Additional file 5: Table S1. The histological examination of CAG lesions through hematoxylin and eosin (HE) staining revealed the presence of pathological changes (Fig. 1B).

After the normalization of read counts and sample integration, we performed dimensionality reduction and unsupervised cell clustering and finally obtained 12 cell clusters in this dataset. On the basis of the expression of canonical marker genes and the top differentially expressed genes (DEGs) of these clusters, we further annotated these clusters as T cells, B cells, plasma cells, myeloid cells, mast cells, fibroblasts, pericytes, endothelial cells, and epithelial cells (Fig. 1C and E). We then separated cells by disease conditions and found that the cell types identified in the two groups were similar, and no cell type specific to a certain disease stage was found in our study (Fig. 1D). In our dataset, we detected an increased abundance of T cells and epithelial cells and a decreased abundance of fibroblasts, endothelial cells, mast cells, myeloid cells and pericytes in CAG samples (Fig. 1F).

### Identification of altered atrophic gastric epithelial cell phenotypes

**Fig. 2:**
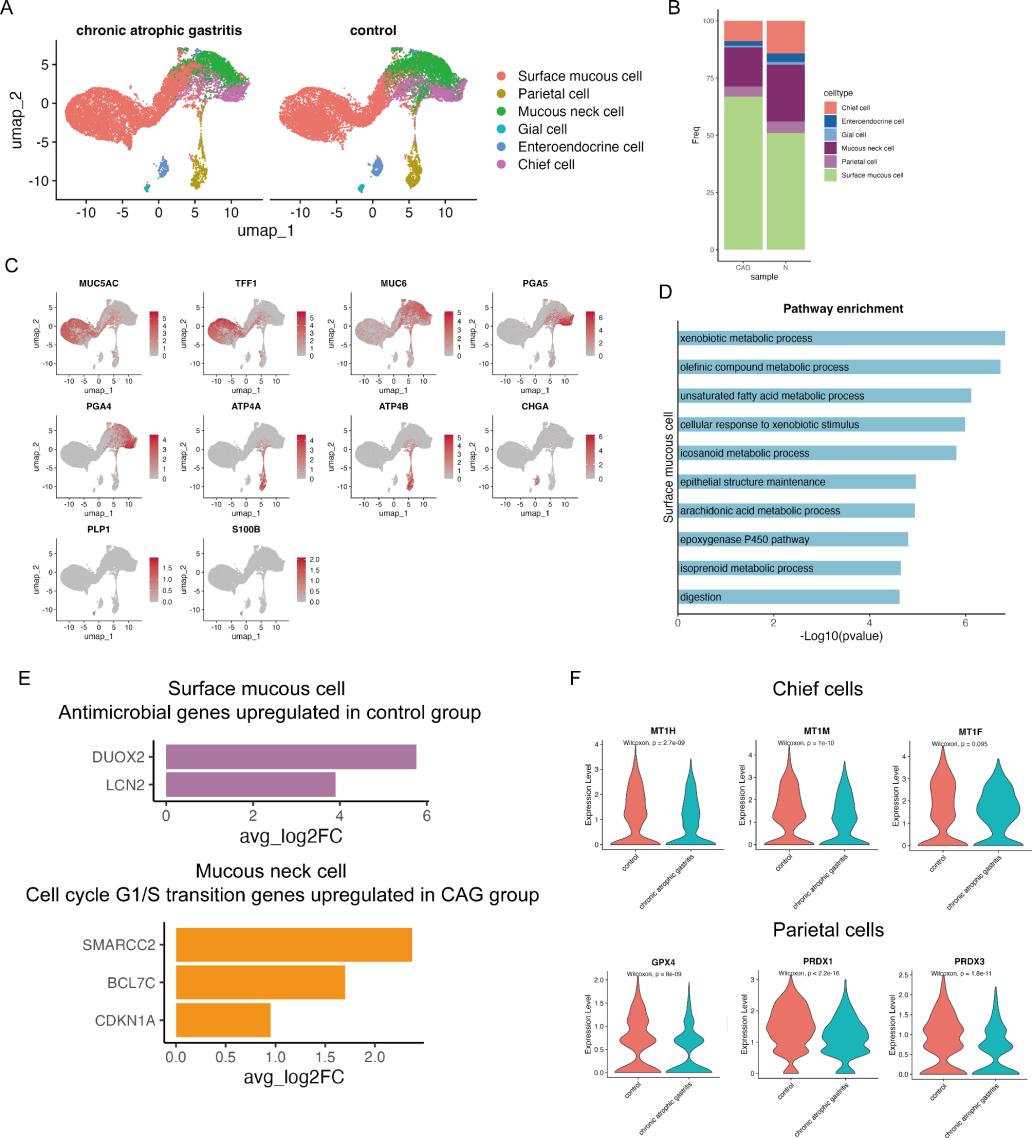
Altered transcription states in different epithelial cell subtypes. (**A**) UMAP plot of epithelial cells, color-coded by cell type. (**B**) Feature plot color-coded for expression (gray to red) of marker genes for the clusters as indicated. (**C**) Proportions of epithelial cell subclusters in CAG and control samples. (**D**) Selected pathways enriched in surface mucous cells versus other cell epithelial cell subtypes. (**E**) Bar plots showing upregulated antimicrobial genes in the control group and upregulated cell cycle G1/S transition genes in the CAG group. (**F**) Violin plots showing the expression levels of detoxification genes in chief cells and parietal cells between the CAG and control samples. P values were calculated via the two-sided Wilcoxon test.

We observed high cellular heterogeneity in the gastric epithelial cells. Projected into the two-dimensional principal component analysis (PCA) space, the gastric epithelial cells were further clustered into 6 gastric lineages, namely, surface mucous cells (*MUC5AC* and *TFF1*), mucous neck cells (*MUC6*), chief cells (*PGA4* and *PGA5*), parietal cells (*ATP4A* and *ATP4B*), enteroendocrine cells (*CHGA*), and glial cells (*PLP1* and *S100B*) [10, 24] (Fig. 2A and C). We observed that the cell types found under the two conditions were the same. However, the proportions of cells in these two conditions were different. In both groups, the major cell population was surface mucous cells, followed by mucous neck cells, chief cells, parietal cells, enteroendocrine cells and glial cells. As expected, the CAG group showed loss of chief cells. The proportion of mucous neck cells also decreased in the CAG group, and surface mucous cells emerged in the CAG group (Fig. 2B). This finding suggested that the expansion of surface mucous cells may occur in CAG lesions. We further investigated the potential function of surface mucous cells and found that surface mucous cells were enriched in the digestion pathway and maintenance of the gastrointestinal epithelium; more importantly, their expressed genes were involved in different metabolic processes, including xenobiotic metabolic processes and olefinic compound metabolic processes (Additional file 7: Table S2). These findings indicate that surface mucous cells expand to undergo various metabolic processes to maintain normal gastric functions under CAG conditions.

To illustrate the changes of the expression program in each epithelial cell subtype during progression to atrophic gastritis, we focused on the differences in the gene expression of each epithelial cell subtype between the two conditions. Interestingly, we observed downregulation of the antimicrobial molecules *DUOX2* and *LCN2* in the surface mucous cells of the CAG group, suggesting an attenuated antimicrobial defense of these cells in the CAG group. We also identified several upregulated genes involved in the cell cycle G1/S phase transition in mucous neck cells, including *SMARCC2*, *BCL7C*, and *CDKN1A*, indicating that mucous neck cells acquired an aberrant cell proliferation phenotype in the atrophic gastritis stage (Fig. 2E). We examined the transcriptional changes in chief cells and parietal cells and found that the metal detoxification genes *MT1H* (*p* = 2.7e^− 09^, Wilcoxon test), *MT1M* (*p* = 1e-^10^, Wilcoxon test) and *MT1F* (*p* = 0.095, Wilcoxon test) were downregulated in chief cells and that the antioxidant genes *GPX4* (*p* = 8e^− 09^, Wilcoxon test), *PRDX1* (*p* < 2.2e^− 16^, Wilcoxon test) and *PRDX3* (*p* = 1.8e^− 11^, Wilcoxon test) were downregulated in parietal cells, indicating an impaired epithelial detoxification system in these two cell subpopulations (Fig. 2F).

We generated atrophic gastritis susceptibility gene scores on the basis of the gene set provided by atrophic gastritis in the Harmonizome 3.0 database (https://maayanlab.cloud/Harmonizome/). We found that the score was greater in the epithelial compartment of the gastric mucosa (Additional file 1: Figure S1A). We further examined the atrophic gastritis susceptibility gene score in different epithelial subtypes and found that the susceptibility gene score was greater in surface mucous cells, parietal cells, mucous neck cells and chief cells (Additional file 1: Figure S1B). More importantly, the susceptibility gene score was significantly higher in the chief cells and mucous neck cells of CAG tissue than in those of normal gastric tissue (Additional file 1: Figure S1C), indicating that the atrophic gastritis susceptibility genes may be related to the aberrant alteration of the expression program in chief cells and mucous neck cells during progression to CAG. Furthermore, to investigate the dynamic changes of cancer-related hallmarks in the CAG group, we generated a gene signature score for the cell cycle and pEMT, which was previously reported as a malignant cell transcription program [25]. Compared with those in neighboring control samples, the pEMT program was significantly elevated in surface mucous cells, mucous neck cells, glial cells, enteroendocrine cells and chief cells of CAG lesions. Compared with those of neighboring control samples, the cycle program was also significantly elevated in surface mucous cells, mucous neck cells, enteroendocrine cells and chief cells of CAG lesions (Additional file 1: Figure S1D). These findings suggested a gain of the malignant phenotype in the epithelial cells of the CAG group.

### CAG is associated with an increased exhausted T-cell phenotype and a decreased cytotoxic T-cell phenotype

**Fig. 3:**
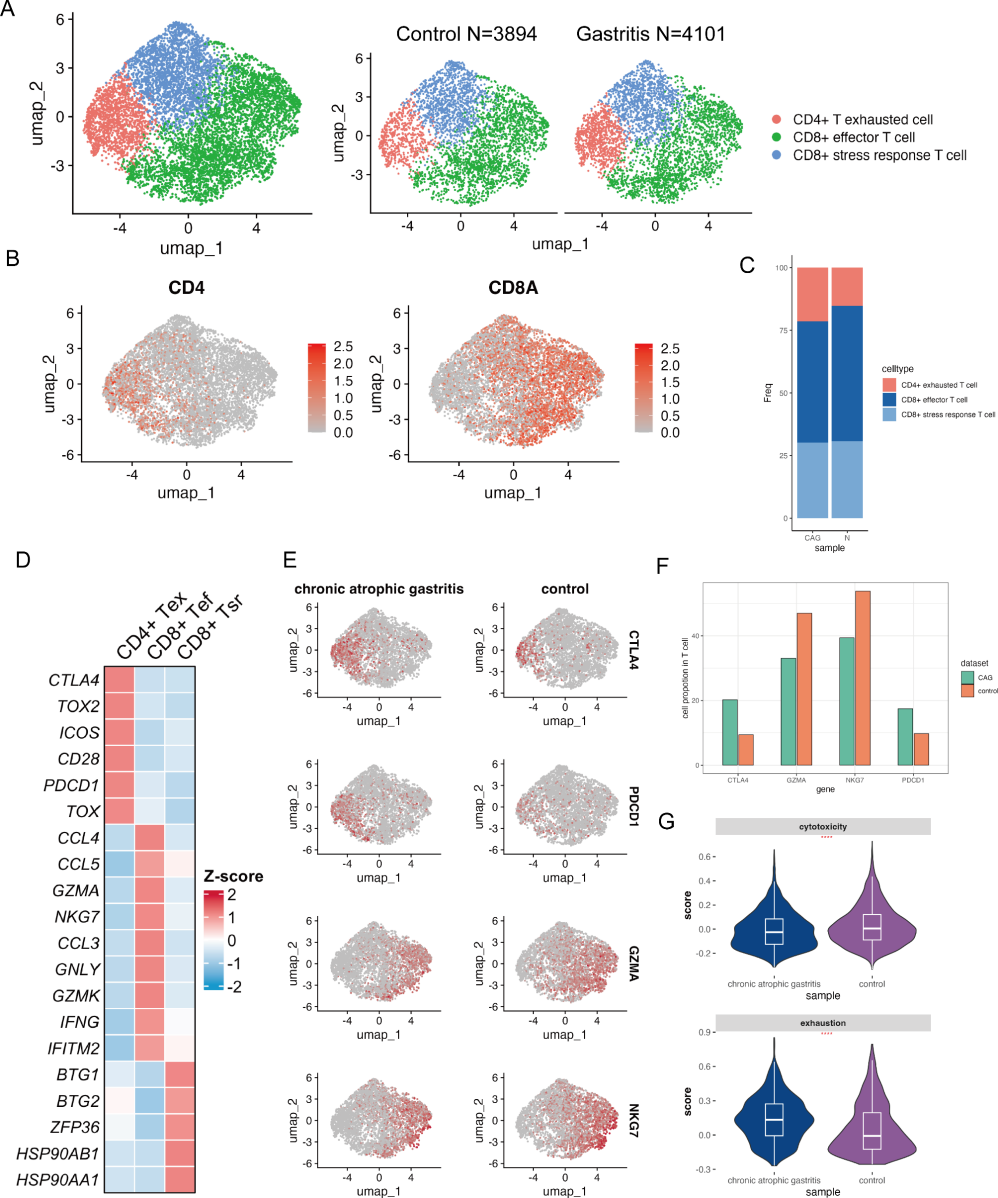
CAG is associated with an increased exhausted T-cell phenotype and a decreased cytotoxic T-cell phenotype. (**A**) UMAP plot of T cells, color-coded by cell type. (**B**) Feature plot showing CD4- and CD8A-expressing T cells. (**C**) Proportions of T-cell subclusters in CAG and control samples. (**D**) Heatmap displaying the expression of selected marker genes for each T-cell subcluster. *CD4*^+^ Tex: exhausted *CD4*^+^ T-cellcell subtype, *CD8*^+^ Tef: *CD8*^+^ T effector cell subtype, *CD8*^+^ Tsr: *CD8*^+^ T stress response. (**E**) Feature plots of representative DEGs between CAG and control samples. (**F**) Proportion of *CTLA4-*, *PDCD1-*, *GZMA-*, and *NKG7*-expressing T cells in CAG and control samples. (**G**) Signature scores of gene sets related to T-cell cytotoxicity in *CD8*^+^ effector T cells and T-cell exhaustion in exhausted CD4 + T cells. P values were calculated via the two-sided Wilcoxon test. ****, *p* < 0.0001

To further evaluate the transcriptional changes in the T-cell population during the development of CAG, we reclassified the T-cell population into three subpopulations according to the specific marker expressed by each cell subtype, namely, *CD4*^+^ exhausted T cells, *CD8*^+^ effector T cells, and *CD8* + stress response T cells (Fig. 3A and B, Additional file 2: Figure S2 A-C). We further compared the distributions of the T-cell subclusters. The percentage of exhausted *CD4*^+^ T cells was greater in the CAG group, the percentage of stress response *CD8*^+^ T cells remained similar under both conditions, and the percentage of *CD8*^+^ effector cells was correspondingly lower, suggesting infiltration of cells with the exhausted phenotype and a reduction in effector functions in the immune compartment of CAG (Fig. 3C). Notably, *CD8*^+^ effector T cells have high expression levels of chemokine genes, including *CCL4*, *CCL5* and *CCL3*, which suggests that *CD8*^+^ effector T cells are a source of immune-recruiting cells that release chemoattractants and promote the immune response [26]. Additionally, *CD8*^+^ effector T cells express high levels of cytotoxic molecules, including *GZMA*, *NKG7*, *GNLY*, *GZMK* [27], the antimicrobial molecule *IFNG* [28] and the antiviral molecule *IFITM2* [29]. As expected, exhausted *CD4*^*+*^ T cells express high levels of the inhibitory molecules *CTLA4*, *TOX2*, *ICOS*, *CD28*, *PDCD1* and *TOX* [27]. We also found a subpopulation of T cells that show a stress response phenotype and that highly express the molecules *BTG1*, *BTG2*, and *ZFP36* to restrain T-cell activation and proliferation [30, 31] (Fig. 3D). In the search for upstream regulatory mechanisms of different T-cell subtypes, we inferred TF activity via the Dorothea algorithm [32, 33]. We observed differences in TF activity among the three T-cell subtypes. TFs with higher activity scores, including *IRF4*, *GATA6* and *STAT3*, in the exhausted *CD4*^+^ T-cell subtype were observed to have low activity in the *CD8*^+^ T effector cell subtype. However, the *CD8*^+^ T effector cell subtypes had high TF activity scores for *IRF1*, *IRF3* and *STAT4* (Additional file 2: Figure S2D). These findings indicate differences in transcription between exhausted *CD4*^+^ T cells and effector *CD8*^+^ T cells. We further compared the TF activity of each T-cell subtype between the CAG and control conditions. We found that the active TFs of each cell subtype under the two conditions were similar, but in the CAG state, exhausted T cells had higher TF activity scores, and effector T cells had lower TF activity scores than did control T cells (Additional file 2: Figure S2E). This result suggested that the transcriptional program related to the exhausted phenotype is more active in the CAG state and that the transcriptional program related to the effector phenotype is more active in the control state.

Next, we conducted differential expression analysis between the two conditions, and higher expression levels of exhaustion-related genes, including *PDCD1* and *CTLA4*, and lower expression levels of the cytotoxic genes *GZMA* and *NKG7* were found in the T cells than in the control cells (Fig. 3E). We also observed infiltration of *PDCD1*^+^ and *CTLA4*^+^ T cells in the tissue microenvironment of CAG lesions. The cell ratio of *PDCD1*^+^ T cells in the CAG group was 0.175; however, in the control group, the ratio decreased to 0.097. The ratio of *CTLA4*^+^ T cells in the CAG group was 0.202; in the control group, the ratio was 0.093. The number of cytotoxic T cells in the tissue microenvironment decreased. The ratio of *NKG7*^+^ T cells was 0.393 in the CAG group and 0.538 in the control group, and the ratio of *GZMA*^+^ T cells was 0.33 in the CAG group and 0.470 in the control group (Fig. 3F). We compared the signature scores between the CAG and non-CAG groups and found that the exhaustion score of exhausted *CD4*^*+*^ cells was significantly elevated in the CAG group, whereas the cytotoxic score of *CD4*^+^ effector cells was significantly decreased in the CAG group (Fig. 3G). These results suggest that the development of atrophic gastritis is associated with an exhausted phenotype and reduced cytotoxic activity of T cells.

### Cellular states of *C1Q*^+^ macrophages and mast cells in CAG

**Fig. 4:**
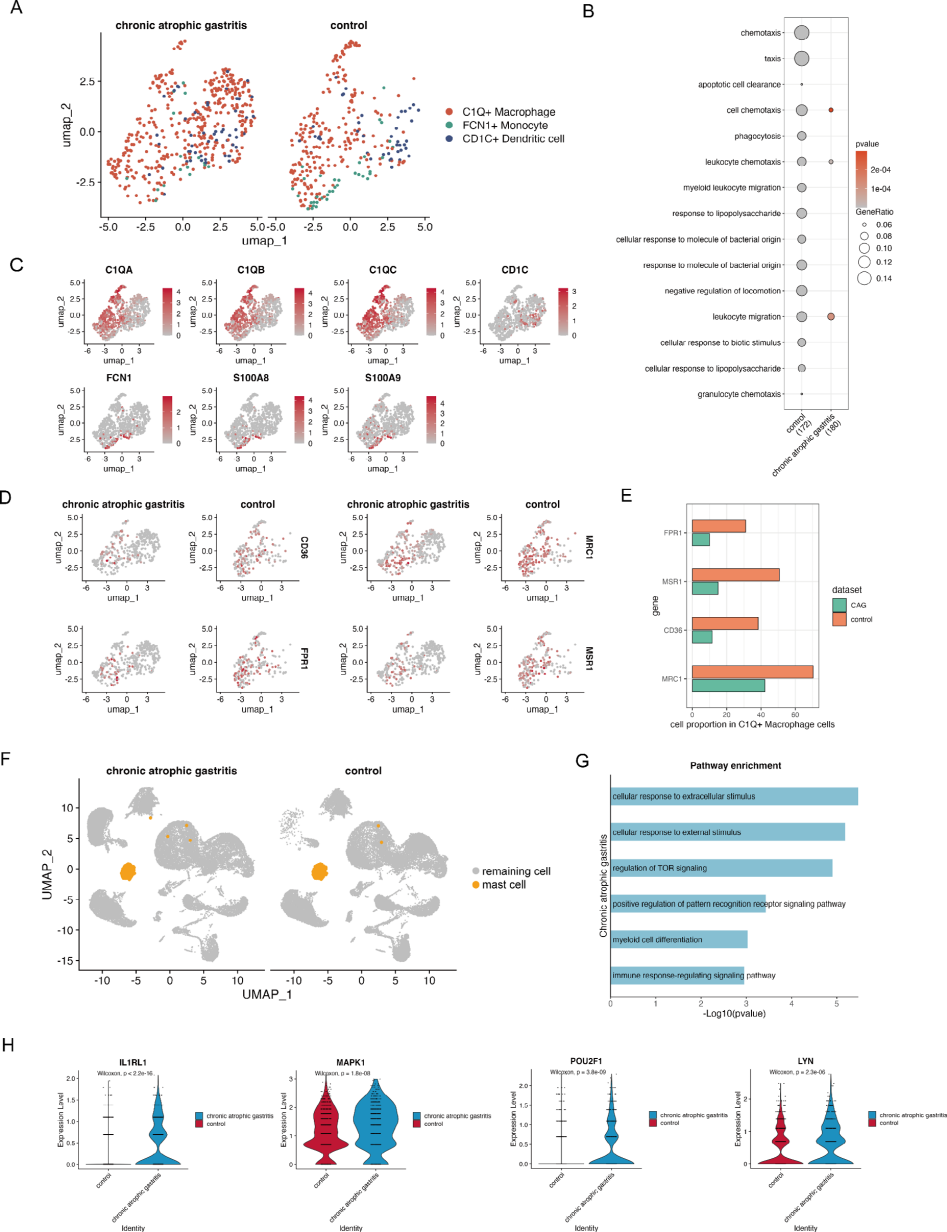
CAG is characterized by reduced phagocytosis and an activated mast cell phenotype. (**A**) UMAP plot of myeloid cells, color-coded by cell type. (**B**) Bubble plot showing significantly enriched pathways of myeloid cells in CAG and control samples. (**C**) Feature plot color-coded for expression (gray to red) of marker genes for the clusters as indicated. (**D**) Feature plots of representative DEGs between CAG and control samples. (**E**) Proportion of *FPR1-*, *MSR1-*, *CD36-*, and *MRC1*-expressing macrophages in CAG and control samples. (**F**) UMAP plot of mast cells. (**G**) Bar plots showing pathways enriched in mast cells in the CAG group. (**H**) Violin plots showing the differences in the expression levels of mast cell activation genes between CAG and control samples. P values were calculated via the two-sided Wilcoxon test.

We further investigated the potential roles of the innate immune system in CAG. The myeloid cells were further divided into *C1Q*^+^ macrophages, *FCN1*^+^ monocytes and *CD1C*^+^ dendritic cells on the basis of the expression of canonical cell markers (Fig. 4A and C). To systematically study the functional implications of myeloid cells in CAG, we identified DEGs and further subjected them to GO analysis, which revealed a striking difference in pathway enrichment between the CAG group and the control group. Cell chemotaxis, apoptotic cell clearance, leukocyte migration, phagocytosis, the cellular response to lipopolysaccharide, and the cellular response to molecules of bacterial origin were observed to have high pathway activity in the control group (Fig. 4B, Additional file 10: Table S5). Specifically, the reduced expression of *MRC1*, *FPR1*, *CD36*, and *MSR1* in *C1Q*^+^ macrophages in the CAG group indicated that the phagocytic activity of *C1Q*^+^ macrophages in the CAG group was lower than that in the control group (Fig. 4D and E). Next, we investigated the pattern recognition system and chemokine system of *C1Q*^+^ macrophages in CAG. We generated gene scores for Toll-like receptors and chemokines and found decreased expression levels of TLR family molecules, including *TLR4* and *TLR2*, and chemokine molecules, including *CCL3*, *CCL4* and *CXCL2*, in *C1Q*^+^ macrophages from chronic atrophic gastric mucosa (Additional file 3: Figure S3A). Moreover, we investigated the expression of Toll-like receptors in atrophic mucosa and found that the main Toll-like receptors expressed in *C1Q*^+^ macrophages from CAG lesions and neighboring control tissues were *TLR1*, *TLR2*, *TLR4*, *TLR5*, and *TLR7* (Additional file 3: Figure S3B, S3C). We evaluated the expression levels of matrix metalloproteinases (MMPs) and tissue inhibitors of metalloproteinases (TIMPs) in *C1Q*^+^ macrophages. We found that in CAG lesions, *C1Q*^+^ macrophages expressed mainly *MMP9*, *MMP12*, *MMP14*, *MMP19*, *MMP2*, *MMP25*, *TIMP2* and *TMP2* (Additional file 3: Figure S3D), which suggests that under inflammatory conditions, *C1Q*^+^ macrophages express certain MMPs to degrade the extracellular matrix (ECM) to recruit other inflammatory immune cells. The expression of TIMPs indicates that in CAG lesions, *C1Q*^+^ macrophages can express molecules that block the degradation of the ECM to regulate the chronic inflammatory response.

To gain further insights into the metabolic pathway activity of *C1Q*^+^ macrophages in nonatrophic and atrophic samples, we used the scMetabolism package to systematically quantify metabolic activity in our scRNA-seq data. Interestingly, we found that the activity of multiple metabolic pathways was reduced in atrophic samples (Additional file 3: Figure S3E). We found that nitrogen metabolic activity was significantly increased in *MRC1*^+^ macrophages, *MSR1*^+^ macrophages, *FPR1*^+^ macrophages and *CD36*^+^ macrophages and decreased in atrophic samples. Glycerophospholipid metabolic activity was significantly increased in *MSR1*^+^ macrophages, *FPR1*^+^ macrophages and *CD36*^+^ macrophages and decreased in atrophic samples (Additional file 3: Figure S3F). Phagocytosis is the critical process by which macrophages ingest and eliminate pathogens and requires dynamic changes in plasma membrane fusion and fission. Lipid synthesis has been linked to increased phagocytosis [34]. Our data revealed infiltration of *LDLR*^*+*^ macrophages in CAG lesions (Additional file 3: Figure S3G). Overall, we concluded that the *C1Q*^+^ macrophages in atrophic gastric mucosa were less active in terms of their cellular metabolism, which may be associated with a decreased phagocytic phenotype. Nitrogen metabolic activity and glycerophospholipid metabolism are decreased in atrophic gastric mucosa to limit the phagocytosis of *C1Q*^+^ macrophages.

Mast cells constitute a major sensory arm of the innate immune system. In both the normal control group and the CAG group, we detected infiltration of mast cells (Fig. 4F). We further compared the DEGs. GO analysis revealed that mast cells in the CAG group mainly expressed genes involved in the cellular response to extracellular stimulus, regulation of TOR signaling, positive regulation of the pattern recognition receptor signaling pathway, myeloid differentiation and the immune response regulation signaling pathway (Fig. 4G, Additional file 11: Table S6). In addition, several mast activation-related markers, such as *IL1RL1* (*p* < 2.2e^− 16^, Wilcoxon test), *MAPK1* (*p* = 1.8e^− 08^, Wilcoxon test), *POUF1* (*p* < 3.8e^− 09^, Wilcoxon test) and *LYN* (*p* = 2.3e^− 06^, Wilcoxon test) [35], were upregulated in CAG lesions (Fig. 4H). These results suggest that mast cells increase the active sensory ability in CAG conditions.

### Functional roles of different fibroblast subpopulations in CAG lesions

**Fig. 5:**
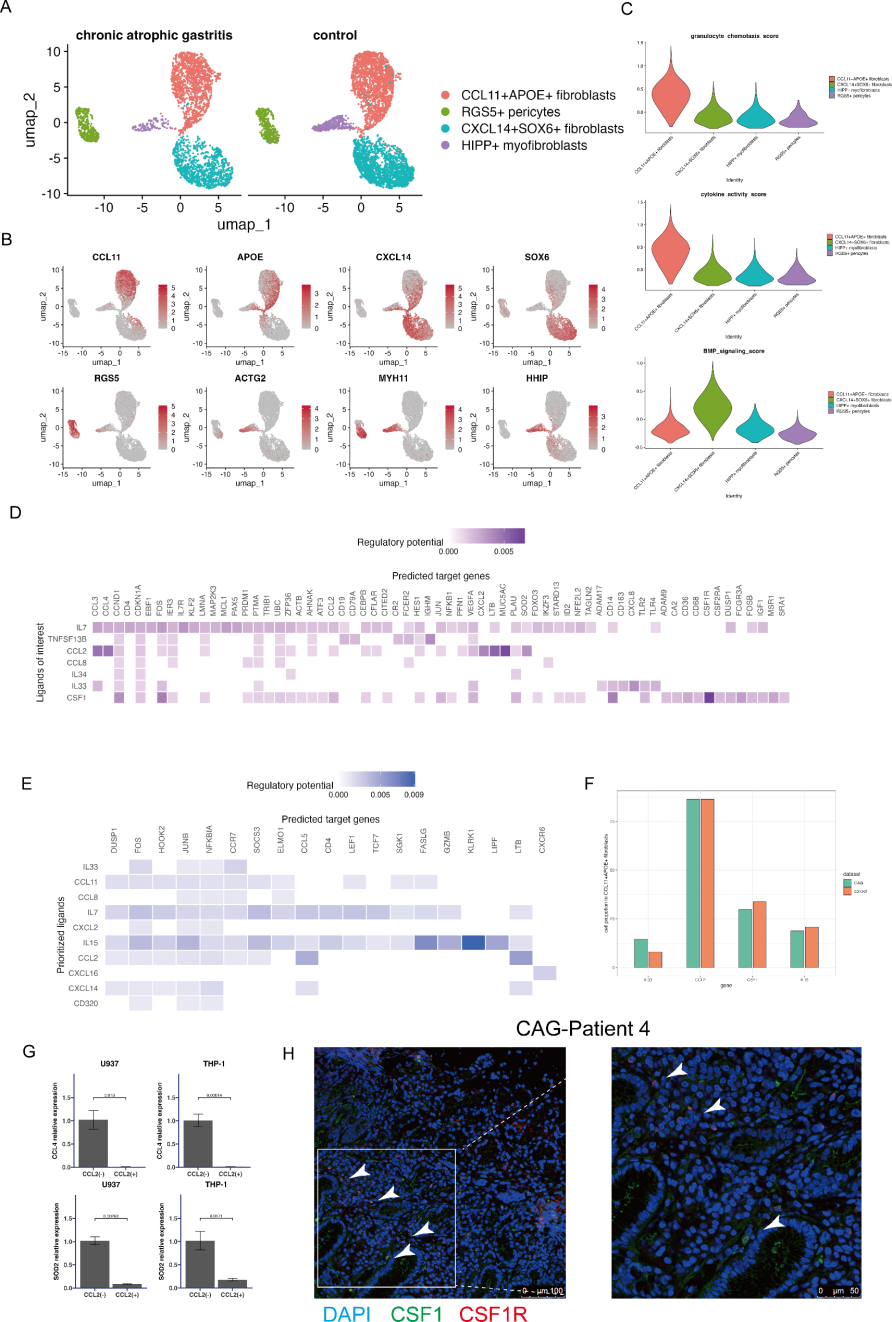
Regulatory potential of different fibroblast subsets on *C1Q*^+^ macrophages and *CD8*^+^ effector cells. (**A**) UMAP plot of fibroblasts and pericytes, color-coded by cell type. (**B**) Feature plot color-coded for expression (gray to red) of marker genes for the clusters as indicated. (**C**) Violin plots showing enrichment of cytokine activity, granulocyte chemotaxis, and BMP signaling pathways in different fibroblast subtypes and pericytes. (**D**) Heatmap showing the cellular regulatory potential of *CCL11*^+^*APOE*^+^ fibroblasts and *C1Q*^+^ macrophages. (**E**) Heatmap showing the cellular regulatory potential of *CCL11*^+^*APOE*^+^ fibroblasts and *CD8*^+^ effector T cells. (**F**) Differences in the expression of the chemokine *CCL2* and the cytokines *IL33* and *IL15* in *CCL11*^+^*APOE*^+^ fibroblasts from CAG and control samples. (**G**) Boxplot showing *CCL4* and *SOD2* expression levels detected by RT‒PCR in CCL2-treated THP-1 and U937 cells. P values were calculated via the two-sided Wilcoxon test. (**H**) mIHC staining of the CSF1-CSF1R interaction in the CAG tissue microenvironment. CAG tissues were stained with CSF1 (green), CSF1R (red) and DAPI for DNA (blue).

Fibroblasts play an important role in regulating inflammation. In our research, we discovered four fibroblast clusters: *CCL11*^+^*APOE*^+^ fibroblasts, *CXCL14*^+^*SOX6*^+^ fibroblasts, *HIPP*^+^ myofibroblasts, and *RGS5*^+^ pericytes (Fig. 5A and B, Additional file 4: Figure S4A). *CCL11*^+^*APOE*^+^ fibroblasts and *CXCL14*^+^*SOX6*^+^ fibroblasts were the major fibroblast populations found in our samples. The ratio of *CCL11*^+^*APOE*^+^ fibroblasts to *RGS5*^+^ pericytes increased in the CAG stage, whereas the ratio of *CXCL14*^+^*SOX6*^+^ fibroblasts to *HIPP*^+^ myofibroblasts decreased in the CAG stage (Additional file 4: Figure S4B). We further investigated the potential functions of these fibroblast subtypes. On the basis of the markers and enrichment analysis of these two fibroblast subtypes, we discovered that *CCL11*^+^*APOE*^+^ fibroblasts express genes involved in the regulation of immune cell recruitment and the expression of cytokines (*CCL2*, *CXCL1*, *IL33*, and *IL34*) and hence exhibit increased cytokine activity and cellular chemotaxis. Moreover, *CXCL14*^+^*SOX6*^+^ fibroblasts presented elevated BMP signaling activity (Fig. 5C). To comprehensively evaluate the immunological properties of different fibroblast subsets, we evaluated the expression levels of chemokines and cytokines in the CAG group and found that *CCL2*, *CCL8*, *CCL11*, *CXCL1*, *CXCL2*, *CXCL3*, *CXCL12*, *CXCL16*, and *CXCL17* are the main chemokines expressed in *CCL11*^+^*APOE*^+^ fibroblasts and that *CXCL14* and *CXCL12* are the main chemokines expressed in *CXCL14*^+^*SOX6*^+^ fibroblasts; *IL7*, *IL15*, *IL33*, *IL16*, *IL32*, and *IL34* are the main cytokines expressed in *CCL11*^+^*APOE*^+^ fibroblasts; and *IL15* and *IL32* are the main cytokines expressed in *CXCL14*^+^*SOX6*^+^ fibroblasts (Additional file 4: Figure S4C).

### *CCL11*^+^*APOE*^+^ fibroblasts have regulatory effects on *C1Q*^+^ macrophages and *CD8*^+^ effector T cells

We further explored the interactions of *CCL11*^+^*APOE*^+^ fibroblast subpopulations with other immune subtypes in CAG lesions. We used NicheNet to infer ligand-target regulatory potential with a prior model. Focusing on ligands of interest, we observed that fibroblasts expressing *CCL2* displayed high regulatory potential with chemokine molecules expressed by *C1Q*^+^ macrophages, including *CCL3*, *CCL4* and *CXCL2*, indicating that these fibroblast subpopulations regulate the immune recruitment ability of *C1Q*^+^ macrophages [36]. Specifically, the CCL2 ligand likely regulates the superoxide dismutase SOD2 (Fig. 5D). In addition, we found that *IL33*-expressing fibroblasts exert regulatory effects on the innate immune system by regulating Toll-like receptor signaling pathways (*TLR2*, *TLR4* and *LY96*) and scavenger receptors (*CD14* and *CD163*). *CSF1*-expressing fibroblasts may regulate *CD14*, *TLR2*, *CD36*, *CSF1R*, *CSF2RA*, and *MSR1* on *C1Q*^+^ macrophages (Fig. 5D). We also observed strong regulatory effects of *IL15* on *FASLG*, *GZMB* and *KLRK1*, which are well characterized cytotoxicity-related genes, between fibroblasts and *CD8*^+^ effector T cells (Fig. 5E), suggesting that *IL15*-expressing fibroblasts play active roles in regulating T-cell cytotoxicity. We found that both *CCL2*^+^ fibroblasts and *IL15*^+^ fibroblasts infiltrated the microenvironment of the CAG and control samples, that *IL33*^+^ fibroblasts emerged in the CAG samples, and that the number of *CSF1*^+^ fibroblasts slightly decreased in the CAG samples (Fig. 5F). We further conducted ex vivo CCL2 stimulation experiments to verify the ability of *CCL2*-secreting fibroblasts to regulate *C1Q*^+^ macrophages. We found that *CCL4* and *SOD2* expression was strongly inhibited by exogenous CCL2 stimulation (Fig. 5G). Our data revealed that the specific receptor of CCL4, CCR5, was expressed mainly in *CD8*^+^ effector cells (Additional file 4: Figure S4D). Here, we found that CCL2 may limit the infiltration of *CD8*^+^ effector cells via the downregulation of *CCL4*. SOD2 can control ROS, and downregulation of SOD2 may promote macrophage-mediated antimicrobial activity [37]. In addition, we verified the direct interaction between CSF1 and CSF1R via multicolor IHC staining of CAG lesion sections (Fig. 5H, Additional file 5: Figure S5). Therefore, we conclude that, on the basis of the gene expression of fibroblasts and predicted target genes, *CSF1*^*+*^, *CCL2*^+^, *IL33*^+^, and *IL15*^+^ fibroblasts might have persistent effects on regulating the inflammatory response of *C1Q*^+^ macrophages and the cytotoxicity of *CD8*^+^ effector cells in the development of CAG.

### ASPN is an early upregulated marker in the CAG stage of gastric tumorigenesis

**Fig. 6:**
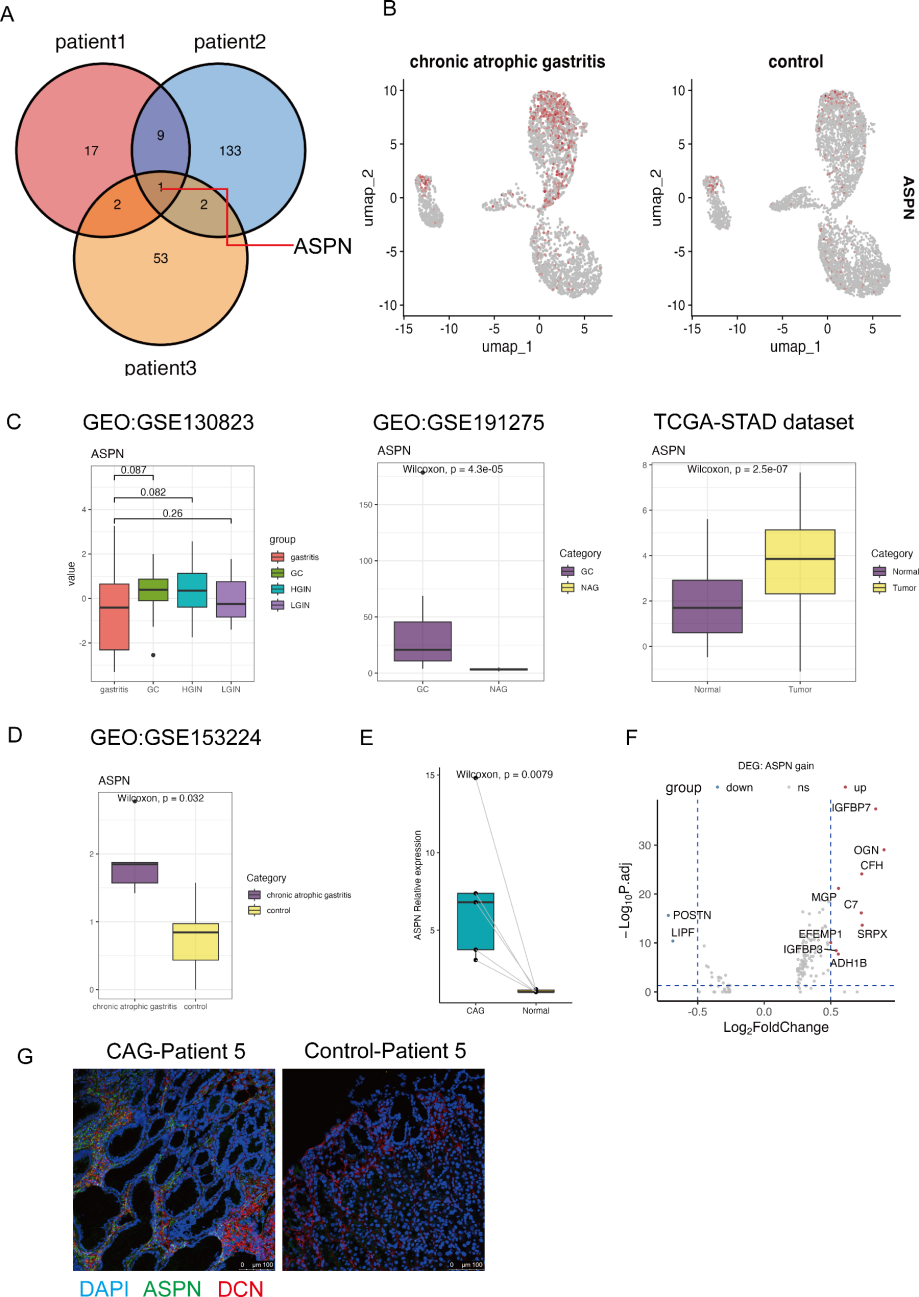
ASPN is an early upregulated marker in the CAG stage of gastric tumorigenesis. (**A**) Venn plot of conservatively upregulated markers, color-coded by patient. (**B**) Feature plot of ASPN gene expression (gray to red) in fibroblast clusters; CAG: left panel; control: right panel. (**C**) Boxplots showing ASPN expression levels between different disease conditions in public datasets (GSE130823, GSE191275, TCGA-STAD dataset). (**D**) Boxplot showing ASPN expression levels between different disease conditions in GSE153224. (**E**) Boxplot showing ASPN expression levels detected by RT‒PCR in CAG and corresponding control samples (*n* = 5). P values were calculated via the two-sided Wilcoxon test. (**F**) Volcano plot showing the DEGs between *APSN*-positive *CCL11*^+^*APOE*^+^ fibroblasts and *ASPN*-negative *CCL11*^+^*APOE*^+^ fibroblasts. (**G**) Representative mIHC staining of ASPN. CAG and control tissues were stained with DCN (red), ASPN (green), and DAPI for DNA (blue)

We separately analyzed the DEGs of *CCL11*^+^*APOE*^+^ fibroblasts and found that the conserved marker *ASPN* was upregulated in CAG samples from three patients (Fig. 6A and B). We additionally download public datasets to conduct external validation. In the GSE130823 dataset, *ASPN* was upregulated in HGIN and GC samples compared with gastritis samples (HGIN: *p* = 0.082, Wilcoxon test; GC: *p* = 0.087, Wilcoxon test). In the GSE191275 dataset, *ASPN* was significantly upregulated in GC patients compared with NAG patients (*p* = 4.3e^− 05^, Wilcoxon test). In the TCGA stomach adenocarcinoma dataset, *ASPN* was significantly upregulated in tumor samples compared with normal samples (*p* = 2.5e^− 07^, Wilcoxon test) (Fig. 6C). In the GSE153224 dataset, *ASPN* was significantly upregulated in CAG samples (*p* = 0.032, Wilcoxon test) (Fig. 6D). qPCR confirmed that the expression of *ASPN* was significantly greater in the CAG samples than in the control samples (*p* = 0.0079, Wilcoxon test, *n* = 5) (Fig. 6E). Immunostaining experiments revealed that APSN was expressed in the stromal region of CAG tissues (Fig. 6G). We concluded that *CCL11*^+^*APOE*^+^ fibroblast-secreted ASPN is an early upregulated marker in the CAG stage of gastric tumorigenesis. We further conducted differential gene expression analysis between *ASPN*^+^*CCL11*^+^*APOE*^+^ fibroblasts and *ASPN*^−^*CCL11*^+^*APOE*^+^ fibroblasts to explore the potential functional alterations of *ASPN*^+^*CCL11*^+^*APOE*^+^ fibroblasts in CAG lesions and found that *ASPN*^+^*CCL11*^+^*APOE*^+^ fibroblasts upregulated *IGFBP7*, *IGFBP3*, *SPRX*, and *ADH1B* (Fig. 6F).

## Discussion

Elucidation of the intricate cellular changes, including the epithelial phenotype and immune cell infiltration, that drive the progression of normal mucosa to atrophic mucosa is indispensable for elucidation of critical events involved in the atrophic mucosa and early intervention in some potential targets. In this study, we examined the changes in different epithelial cell lineages and immune populations associated with the development of CAG. These findings provide new insights for identifying new potential targets that may protect mucosa integrity.

*H. pylori* is the predominant etiological agent of gastritis and disrupts the integrity of the gastric mucosa barrier through various pathogenic mechanisms [38]. Persistent infection by *H. pylori* induces uncontrolled innate and adaptive immune responses, creating a chronically inflamed environment in the gastric mucosa that favors the growth of bacteria [38]. Previous studies have reported that DUOX2 is involved in the sentinel response in mammalian innate defense against potential external threats to the epithelial barrier. The H_2_O_2_ generated by DUOX2 prevents *Helicobacter felis* colonization within the gastric mucus layer [39]. LCN2 attenuates bacterial growth by binding and sequestering iron-scavenging siderophores to obstruct bacterial iron acquisition [40]. In our study, several antimicrobial molecules, such as *DUOX2* and *LCN2*, were downregulated in surface mucous cells under CAG conditions. More importantly, these expression changes were specific to surface mucous cells, indicating that disruption of the bacterial defense system of surface mucous cells may increase susceptibility to bacterial infection and hence contribute to the development of CAG.

In this study, we compared the expression states of different epithelial cell subpopulations, and a notable increase in aberrant cell cycle activity in mucous neck cells was observed in atrophic gastric epithelial tissues. Genes related to the G1/S cell cycle transition, including *SMARCC2*, *BCL7C* and *CDKN1A*, were upregulated in atrophic mucous neck cells. This malignant phenotype transition provides insight into the target epithelial subpopulation that might assist in early intervention in cancer progression. We observed that chief cells and parietal cells exhibit attenuated detoxification ability, mainly via the downregulation of *MT1H*, *MT1M* and *MT1F* in chief cells and *GPX4*, *PRDX1* and *PRDX3* in parietal cells. Inactivation of GPX4 can induce ferroptosis-related cell death, which results in a nonapoptotic form of cell death with the accumulation of toxic lipid peroxides [41]. Evasion of cancer cell ferroptosis via GPX4 upregulation has been observed during GAC progression. Our data revealed that parietal cells in the atrophic gastritis stage may undergo ferroptosis-related cell death, which may lead to histological atrophy instead of ferroptosis evasion [42]. Metallothioneins (MTs) are a family of metal-binding proteins. These proteins regulate the homeostasis of zinc (Zn) and copper (Cu), mitigate heavy metal poisoning, and alleviate superoxide stress [43]. In *H. pylori*-induced gastritis, ROS are generated by a cytotoxin-associated protein A toxin produced by *H. pylori*, resulting in potentially mutagenic and carcinogenic DNA adducts. MTs can reduce ROS and inhibit cell damage through zinc management [44]. Hydroperoxides may increase the genetic instability of cells. The antioxidant enzymes peroxiredoxins and glutathione peroxidases (GPXs) are components of the cellular defense system that can promptly eliminate accumulated ROS. An imbalance between the excessive generation of hydroperoxides and a reduced level of peroxidases contributes to a deleterious oxidative stress status and ultimately facilitates tumorigenesis [45]. Hence, in the CAG stage, detoxification-related genes are downregulated, which might increase the risk of exposure to inorganic carcinogenic agents in the gastric mucosa and the accumulation of deleterious ROS, hence increasing the risk of the gastric mucosa progressing to a cancerous phenotype.

The development of CAG may be attributed to an inadequate immune response, indicating complex regulation between *H. pylori* and the immune system. We found that the infiltration of *C1Q*^+^ macrophages downregulated the expression of genes related to phagocytosis during progression to CAG, which is a critical component of the innate immune system and is responsible for removing harmful microorganisms or malignantly transformed cells [46]. In the normal gastric mucosa, the innate system has a stronger immune response related to granulocyte chemotaxis, granulocyte migration, phagocytosis, endocytosis and the cellular response to LPS, which maintains a greater ability of innate surveillance to kill abnormal epithelial cells or invading bacteria. However, this innate surveillance mechanism is compromised in atrophic mucosa. We speculated that compromised phagocytosis and granulocyte chemotaxis are important events that facilitate the development of CAG. Macrophages engulf bacteria but cannot kill them, thereby leading to chronic *H. pylori* infection of the gastric mucosa and promoting the malignant transformation of gastric epithelial cells. Another possible bacterial invasion strategy we observed in our CAG single-cell transcriptome data is an immune phenotype shift toward exhausted T cells. The presence of the exhausted T-cell subtype indicates that a moderate regulatory mechanism is involved in the development of CAG. More importantly, the increased expression of the immune checkpoints *CTLA4* and *PDCD1* and decreased expression of *GZMA* and *NKG7* provide insight into the differences in the inflammatory microenvironment between normal mucosa and atrophic mucosa. However, the predominant adaptive immune response remains regulated by *CD8*^+^ effector T cells during progression into CAG, which indicates that the microenvironment still has an overwhelming defense against persistent bacterial infection. However, the promotion of the exhausted phenotype might partially explain the gradual formation of an immunosuppressive microenvironment that favors abnormal cell growth.

Fibroblasts reside in epithelial tissue and possess specialized features to support epithelial cells to maintain barrier integrity and function. In chronic infection, inflammation and cancer, tissue-resident fibroblasts are also emerging as important immune-sentinel cells that regulate local immune responses [47], and their immunological properties range from the maintenance of a potent inflammatory environment during chronic inflammation [48] to the promotion of immunosuppression during malignancy and elimination of infectious agents within tissues [49]. In our dataset, we identified a subpopulation of fibroblasts that express cytokines and chemokines, indicating a potential role in modulating immune cell recruitment and maintaining immune responses. We further used the NicheNet tool and *CCL2* simulation experiments to determine the regulatory potential of these fibroblasts in the tissue microenvironment. We identified a fibroblast subtype expressing high levels of the chemokine *CCL2* that could downregulate *CCL4* expression and *SOD2* expression, which indicates that *CCL4*-driven *CD8*^*+*^ effector T-cell infiltration may be controlled by *CCL2*^*+*^ fibroblasts. The ROS-mediated antimicrobial function of macrophages could be elevated via *CCL2*^*+*^ fibroblasts through the downregulation of SOD2. Notably, we detected direct CSF1‒CSF1R interactions in the stromal regions of CAG lesions. CSF1 controls the growth and differentiation of macrophages. CSF1 signaling has been previously found to be required for the maintenance of macrophage populations in adult mice [50]. In the CAG disease model, fibroblasts can secrete CSF1 to maintain persistent macrophage infiltration, which leads to chronic inflammation. We also concluded that the cytokines IL33 and CSF1 can regulate the innate immune receptors of *C1Q*^+^ macrophages; however, further experiments still need to be conducted to confirm the regulatory potential of these cytokines. IL15 has been previously reported to contribute to tissue protection by promoting the elimination of infected cells [51]. IL15 can increase the cytotoxicity of natural killer cells via the upregulation of NKG2D and cytotoxic effector molecule expression [52]. Here, we discovered that *CCL11*^+^*APOE*^+^ fibroblasts are a source of IL15 that regulates *CD8*^+^ T-cell cytotoxicity. We also found a subpopulation of *ASPN*^+^*CCL11*^+^*APOE*^+^ fibroblasts that were differentially expressed in CAG. *ASPN* expression is upregulated during GC progression. Additionally, this fibroblast subpopulation was associated with increased expression of *IGFBP7*. A previous study reported that cancer-associated fibroblasts can secrete IGFBP7 to promote GC [53]. Whether these cell populations promote gastric cascade development in the atrophic gastritis stage should be further investigated.

However, our research has several limitations that should be acknowledged. The first limitation is that we collected data from only 3 pairs of CAG patients, which may have led to analytical bias. Although we found intriguing and seemingly reasonable transcriptional changes in different epithelial subtypes and immune cells, this small sample size created issues related to the reproducibility of these statistically significant changes as well as whether these findings reflect real-world data. Additional volunteers need to be recruited to increase the statistical power in future research. Another limitation is that patient heterogeneity was underestimated in this study. *H. pylori* infection status and disease stage were not considered. A stratified analysis of *H. pylori* infection should be conducted in future research. We acknowledge that the generalizability and clinical translational value of this study are still limited, and patient heterogeneity and individual differences may result in some conclusions that cannot be drawn in other patients with atrophic gastritis. More importantly, the potential impact of different patient characteristics or disease stages on cellular function has not been addressed. Although we found altered phenotypes involving different epithelial subtypes and immune subtypes, as well as interactions between fibroblasts and immune cells, these findings are mainly based on comprehensive bioinformatics analysis. CSF1-CSF1R has been validated in the FFPE tissue of CAG lesions; however, how such crosstalk may affect the progression of CAG should be investigated in future research. CCL2 has been confirmed to downregulate *SOD2* in macrophages. The regulatory potential of CCL2 on ROS also needs to be verified in future research. Since crosstalk between epithelial and immune/stromal compartments is also essential in the pathogenesis of CAG, we need to focus on the underlying mechanism involved in future research.

In our research, we provide new insights showing that macrophage phagocytic function or the T-cell immune response could be effective in targeting cells for early-stage treatment. Although these findings are intriguing, additional in vitro and in vivo experiments are needed to validate the translation potential of these strategies. We believe that decreased macrophage phagocytic function is one possible reason for the accumulation of aberrantly expressed epithelial cells, which ultimately results in cancer development. Intervention in macrophage phagocytosis by increasing phagocytic signals, for example, increasing CD36 expression, could be a promising strategy for preventing the development of CAG. One potential strategy to invigorate the phagocytic response is the use of nanoparticles packed with small-molecule drugs to drive macrophages toward a more phagocytic phenotype. One benefit of these strategies is that the invading pathogen and cancerous cells are effectively cleared, whereas a harmful side effect could be the immune disaster caused by manipulating these immune cells, which could threaten other organ systems. Another challenge for implementing macrophage-based therapy is the possibility of acquiring M2 phenotypes, leading to a tumor-prone microenvironment when exposed to cancerous cells [54]. Therefore, key cell populations or molecular targets that can reverse the pathogenic status of CAG should be further explored in vivo.

## Conclusions

Our study revealed that the innate immune response was inadequate in CAG, which is characterized by decreased phagocytosis and decreased metabolic activities at the single-cell level. The number of exhausted T cells was increased, the number of cytotoxic T cells was decreased in CAG, the activity of TFs related to exhausted T cells was greater in the CAG state, and cytotoxic T cells were less active in the CAG state. Moreover, mucous surface cells and chief cells may be susceptible epithelial cell populations during CAG progression. The epithelial defense abilities of different epithelial subtypes are compromised by the downregulation of detoxification genes. In conclusion, immune decline and epithelial detoxification decline could be key molecular events that drive CAG progression.

## Electronic supplementary material

Below is the link to the electronic supplementary material.

Supplementary Material 1: Additional file 1: Figure S1. Identification of potentially pathogenic epithelial clusters of CAG. (A) Feature plot showing the expression levels of atrophic gastritis susceptibility genes among different cell subtypes. (B) Violin plots showing the expression levels of atrophic gastritis susceptibility genes in different epithelial subtypes. (C) Violin plots comparing the differences in the gene signature scores of atrophic gastritis susceptibility genes between CAG and control samples. (D) Boxplots comparing the differences in the gene signature scores of pEMT and cycle between CAG and control samples. P values were calculated via the two-sided Wilcoxon test. ****, p < 0.0001; ***, p < 0.001; **, p < 0.01; *, p < 0.05.

Supplementary Material 2: Additional file 2: Figure S2. Expression of classic markers in each T-cell subcluster and inferred TF activities of T-cell subclusters. (A) Bubble plot depicting the average expression levels and cellular fractions of selected stress-related heat shock marker genes in 3 defined T-cell subclusters. (B) Bubble plot depicting the average expression levels and percentages of exhaustion-related marker genes in 3 defined T-cell subclusters. (C) Bubble plot depicting the average expression levels and cellular fractions of effector function-related marker genes in 3 defined T-cell clusters. (D) Heatmap showing TF activity among different T-cell subclusters. (E) Heatmap showing TF activity among different T-cell subclusters in the CAG and control groups. CD4^+^ Tex: exhausted CD4^+^ T-cell subtype, CD8^+^ Tef: CD8^+^ T effector cell subtype, CD8^+^ Tsr: CD8^+^ T stress response.

Supplementary Material 3: Additional file 3: Figure S3. Changes in the expression of TLRs, the chemokine system and metabolic activities in macrophages. (A) Violin plot showing the differences in the gene signature scores of chemokines and Toll-like receptors between CAG and control samples. P values were calculated via the two-sided Wilcoxon test. ****, p < 0.0001. (B) Proportion of TLR-expressing C1Q^+^ macrophages in CAG and control samples. (C) Feature plot showing TLR expression levels in C1Q^+^ macrophages in CAG samples. (D) Feature plot showing the expression levels of MMPs and TIMPs in C1Q^+^ macrophages in CAG samples. (E) Bubble plot showing metabolic activity analysis of C1Q^+^ macrophages between CAG and control samples. The circle size and color darkness both represent the scaled metabolic score. The red on the bubble plot shows the top increased metabolic pathways, and the blue shows the top decreased metabolic pathways. (F)﻿ Boxplots showing the metabolic scores of nitrogen metabolism and glycerophospholipid metabolism. P values were calculated via the two-sided Wilcoxon test. ns, not significant, **, P < 0.01 and ****, P < 0.0001. (G) Feature plot showing LDLR expression levels in C1Q^+^ macrophages between CAG and control samples.

Supplementary Material 4: Additional file 4: Figure S4. Gene expression of different fibroblast subpopulations and changes in the proportions of different fibroblast subpopulations between CAG and control tissues. (A) Heatmap of the top 10 genes of the cluster-defining DEGs among all fibroblasts from the control and chronic astrophic gastritis cell populations. Red on the heatmap shows the most highly upregulated genes, and blue shows the most downregulated genes. (B) Proportions of fibroblast subclusters in CAG and control samples. (C) Chemokine and cytokine molecule expression levels in different fibroblast subclusters of CAG. (D) Feature plot showing CCR5 expression in CD8+ T cells.

Supplementary Material 5: Additional file 5: Figure S5. Representative CSF1-CSF1R interaction in the CAG and control tissue microenvironments. Representative mIHC image of the CSF1-CSF1R interaction. CAG tissue sections were stained with CSF1 (green), CSF1R (red), DCN (purple), and DAPI for DNA (blue).

Supplementary Material 6: Additional file 6: Table S1. Sample information.

Supplementary Material 7: Additional file 7: Table S2. GO analysis of pathway enrichment in surface mucous cells.

Supplementary Material 8: Additional file 8: Table S3. List of genes associated with atrophic gastritis susceptibility.

Supplementary Material 9: Additional file 9: Table S4. Curated gene signatures to calculate scores.

Supplementary Material 10: Additional file 10: Table S5. GO analysis of pathway enrichment in myeloid cells between CAG and control tissues.

Supplementary Material 11: Additional file 11: Table S6. GO analysis of pathway enrichment in mast cells from CAG patients.

## Data Availability

The single-cell sequencing data of this study have been submitted to the Genome Sequence Archive for Human (GSA for humans) database. The accession number is HRA007844.

## Abbreviations

BP: Biological process
CAG: Chronic atrophic gastritis
DEGs: Differentially expressed genes
ECM: Extracellular matrix
GC: Gastric cancer
GO: Gene Ontology
H. pylori: Helicobacter pylori
HE: Hematoxylin and eosin
HGIN: High-grade intraepithelial neoplasia
IHC: Immunohistochemistry
KEGG: Kyoto Encyclopedia of Genes and Genomes
LGIN: Low-grade intraepithelial neoplasia
MTs: Metallothioneins
MMPs: Matrix metalloproteinases
NAG: Nonchronic gastritis
scRNA: seq-Single-cell RNA sequencing
TIMPs: Tissue inhibitors of metalloproteinases
PCA: Principal component analysis
TF: Transcription factor
