# Supplementary figures and images for: Single-cell profiling reveals a reduced epithelial defense system, decreased immune responses and the immune regulatory roles of different fibroblast subpopulations in chronic atrophic gastritis
Source: J Transl Med. 2025 Feb 4;23:159. doi: 10.1186/s12967-025-06150-w (PMC11796052; doi:10.1186/s12967-025-06150-w)

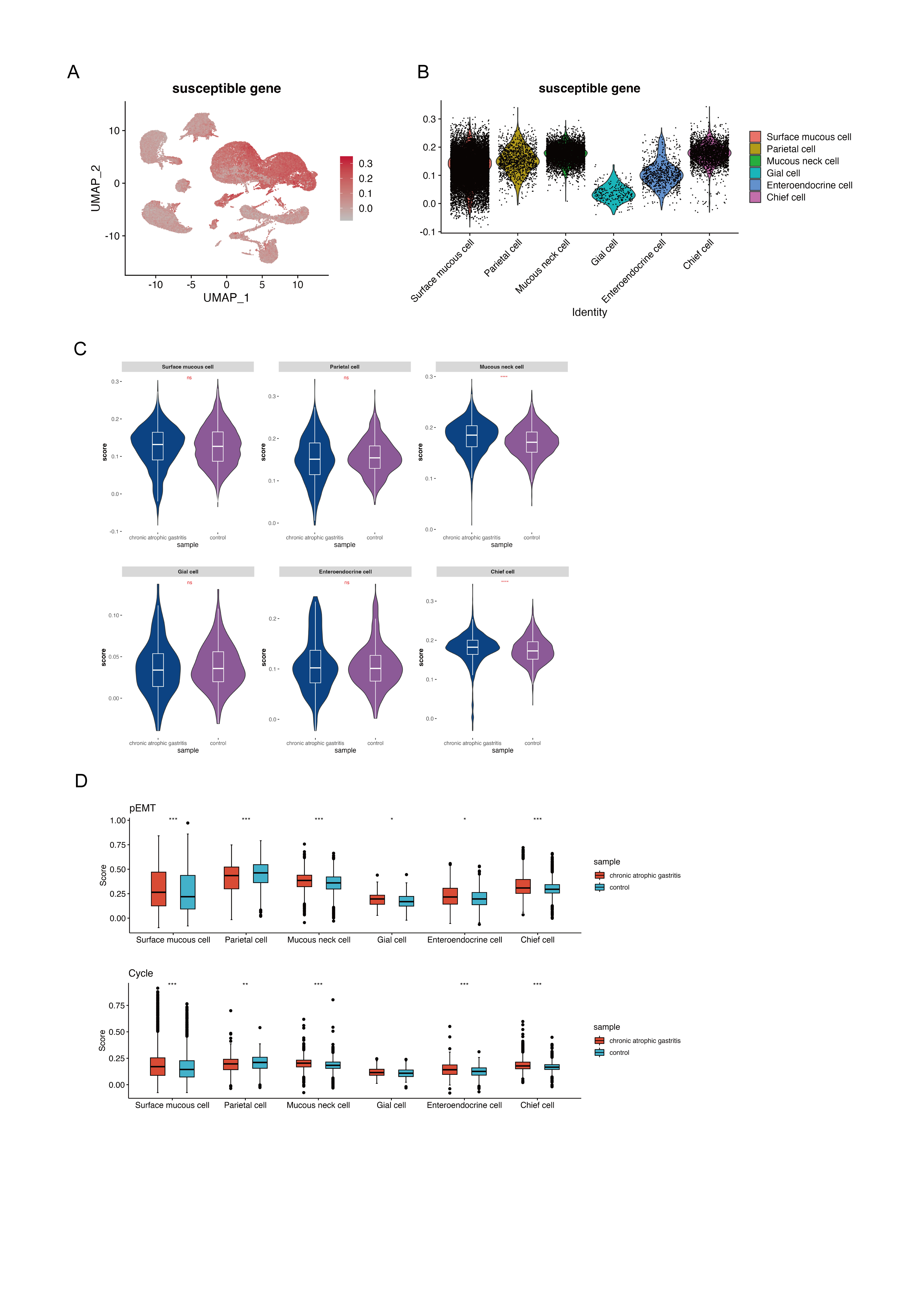

Supplement: Supplementary file 7 — Supplementary Material 7: Additional file 7: Table S2. GO analysis of pathway enrichment in surface mucous cells. [file 12967_2025_6150_MOESM7_ESM.tif]

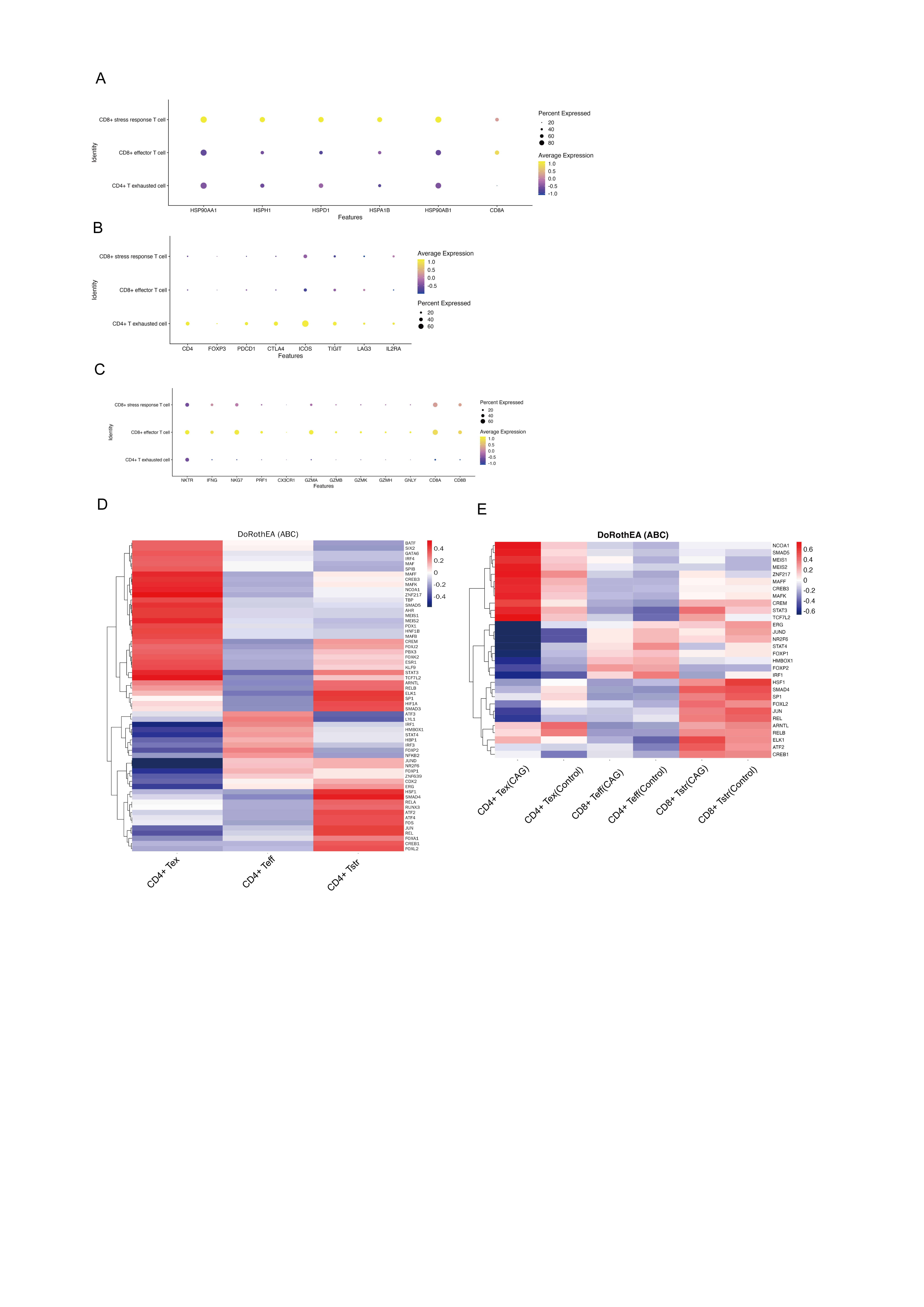

Supplement: Supplementary file 8 — Supplementary Material 8: Additional file 8: Table S3. List of genes associated with atrophic gastritis susceptibility. [file 12967_2025_6150_MOESM8_ESM.tif]

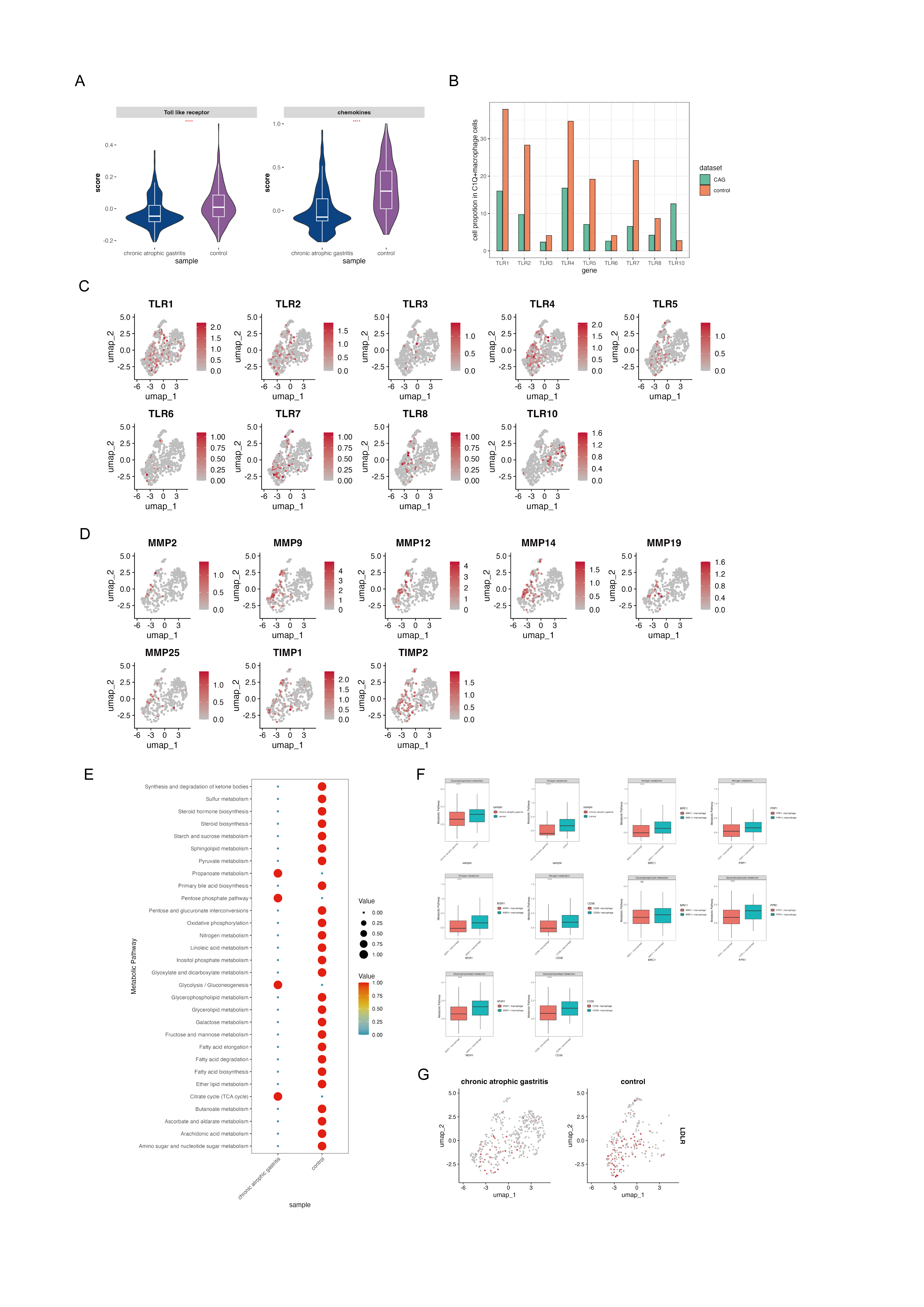

Supplement: Supplementary file 9 — Supplementary Material 9: Additional file 9: Table S4. Curated gene signatures to calculate scores. [file 12967_2025_6150_MOESM9_ESM.tif]

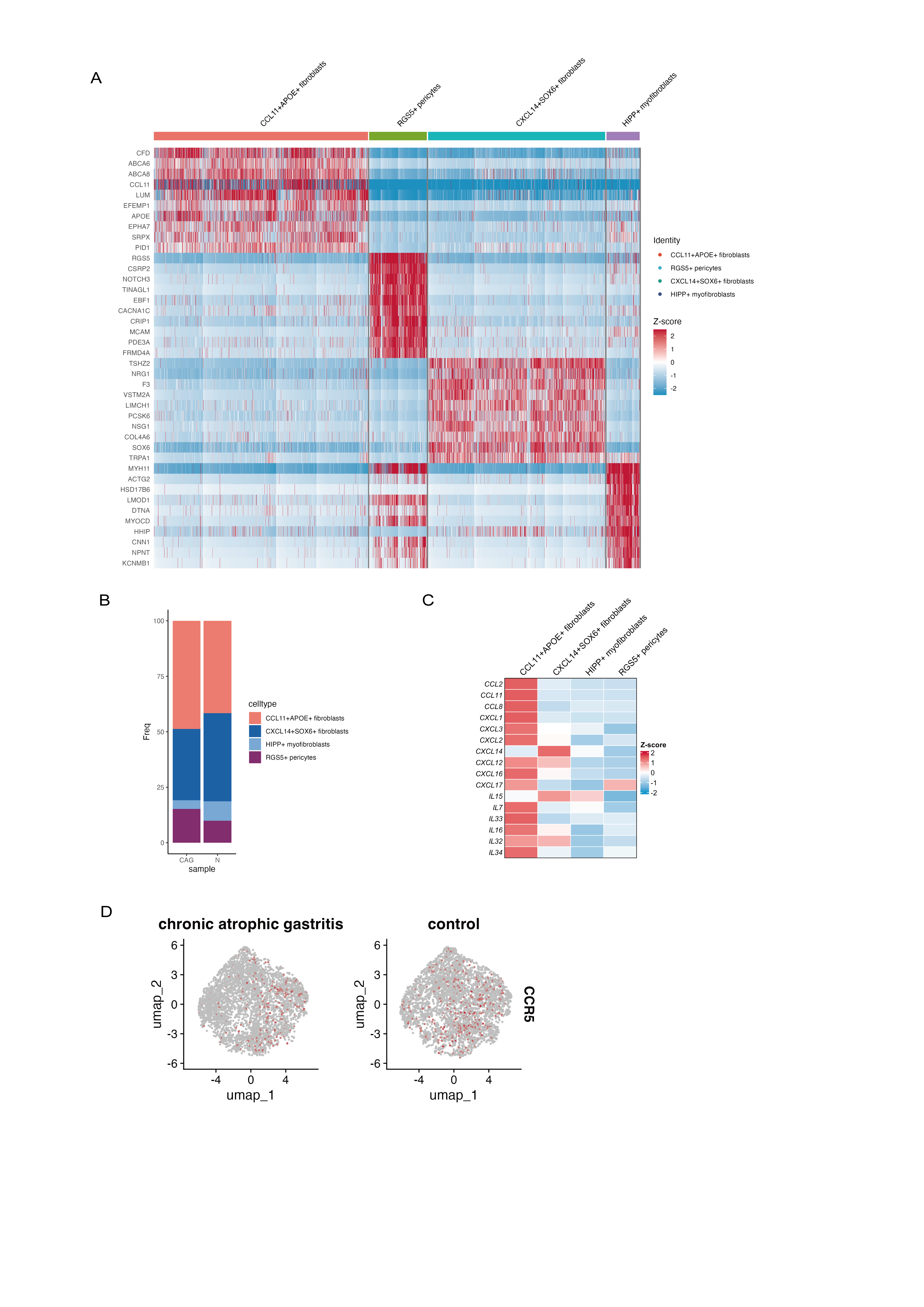

Supplement: Supplementary file 10 — Supplementary Material 10: Additional file 10: Table S5. GO analysis of pathway enrichment in myeloid cells between CAG and control tissues. [file 12967_2025_6150_MOESM10_ESM.tif]

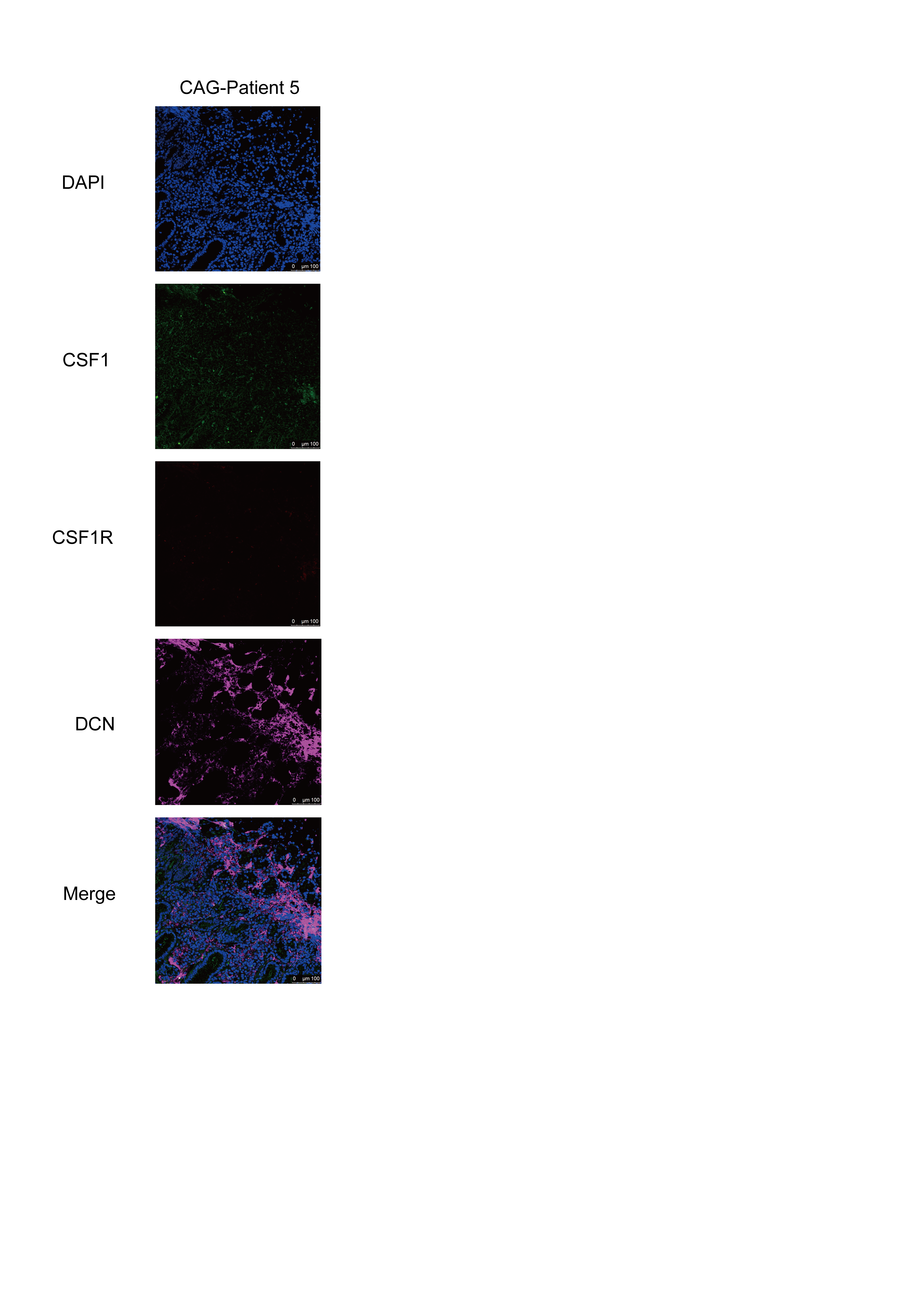

Supplement: Supplementary file 11 — Supplementary Material 11: Additional file 11: Table S6. GO analysis of pathway enrichment in mast cells from CAG patients. [file 12967_2025_6150_MOESM11_ESM.tif]
